# Natural statin derivatives as potential therapy to reduce intestinal fluid loss in cholera

**DOI:** 10.1371/journal.pntd.0010989

**Published:** 2022-12-09

**Authors:** Rattikarn Noitem, Pawin Pongkorpsakol, Chartchai Changsen, Yaowapa Sukpondma, Chittreeya Tansakul, Vatcharin Rukachaisirikul, Chatchai Muanprasat

**Affiliations:** 1 Program in Translational Medicine, Faculty of Medicine Ramathibodi Hospital, Mahidol University, Ratchathewi, Bangkok, Thailand; 2 Chakri Naruebodindra Medical Institute, Faculty of Medicine Ramathibodi Hospital, Mahidol University, Bang Phli, Samut Prakarn, Thailand; 3 Princess Srisavangavadhana College of Medicine, Chulabhorn Royal Academy, Bangkok, Thailand; 4 Department of Microbiology, Faculty of Science, Mahidol University, Ratchathewi, Bangkok, Thailand; 5 Division of Physical Science and Center of Excellence for Innovation in Chemistry, Faculty of Science, Prince of Songkla University, Hat Yai, Songkhla, Thailand; Wayne State University, UNITED STATES

## Abstract

As a leading cause of death in children under 5 years old, secretory diarrheas including cholera are characterized by excessive intestinal fluid secretion driven by enterotoxin-induced cAMP-dependent intestinal chloride transport. This study aimed to identify fungal bioactive metabolites possessing anti-secretory effects against cAMP-dependent chloride secretion in intestinal epithelial cells. Using electrophysiological analyses in human intestinal epithelial (T84) cells, five fungus-derived statin derivatives including α,β-dehydrolovastatin (DHLV), α,β-dehydrodihydromonacolin K, lovastatin, mevastatin and simvastatin were found to inhibit the cAMP-dependent chloride secretion with IC_50_ values of 1.8, 8.9, 11.9, 11.4 and 5 μM, respectively. Being the most potent statin derivatives, DHLV was evaluated for its pharmacological properties including cellular toxicity, mechanism of action, target specificity and *in vivo* efficacy. DHLV at concentrations up to 20 μM did not affect cell viability and barrier integrity of T84 cells. Electrophysiological analyses indicated that DHLV inhibited cystic fibrosis transmembrane conductance regulator (CFTR), a cAMP-dependent apical chloride channel, via mechanisms not involving alteration of intracellular cAMP levels or its negative regulators including AMP-activated protein kinases and protein phosphatases. DHLV had no effect on Na^+^-K^+^ ATPase activities but inhibited Ca^2+^-dependent chloride secretion without affecting intracellular Ca^2+^ levels. Importantly, intraperitoneal (2 mg/kg) and intraluminal (20 μM) injections of DHLV reduced cholera toxin-induced intestinal fluid secretion in mice by 59% and 65%, respectively without affecting baseline intestinal fluid transport. This study identifies natural statin derivatives as novel natural product-derived CFTR inhibitors, which may be beneficial in the treatment of enterotoxin-induced secretory diarrheas including cholera.

## Author summary

Secretory diarrheas including cholera are an important global health problem requiring new therapeutic approaches to reduce associated morbidity and mortality. In this study, we discovered that several natural statin derivatives including lipid-lowering agents inhibited cAMP-dependent chloride secretion with α,β-dehydrolovastatin (DHLV) isolated from the soil-derived fungus *Aspergillus sclerotiorum* PSU-RSPG178 being the most potent compound. DHLV inhibited CFTR chloride channels via mechanisms not involving alteration of intracellular cAMP levels or activation of negative regulators of CFTR functions. Furthermore, DHLV was non-toxic to intestinal epithelial cells and had no effect on Na^+^-K^+^ ATPase activities. *In vivo* administration of DHLV suppressed intestinal fluid secretion in a mouse closed loop model of cholera. Natural statins represent a promising class of compound candidates for the development of anti-secretory therapy of cholera.

## 1. Introduction

Secretory diarrhea is one of the major causes of morbidity and mortality especially in developing countries with the estimated number of 1.6 million deaths annually. It is considered as a third leading cause of death in children under 5 years of age [1]. The common etiologies of secretory diarrheas are intestinal infections with pathogens that produce enterotoxins capable of inducing Cl^-^-driven intestinal fluid secretion [2]. Mainstay therapy of secretory diarrheas is the use of oral rehydration solution (ORS), which replaces fluid loss without affecting severity of diarrheas making it ineffective in patients with severe diarrheas [3,4]. Therefore, there is a need to develop anti-secretory therapy that targets molecular mechanisms determining severity of secretory diarrheas especially ion transport in enterocytes [5].

Ion transport in enterocytes is mediated by several transport proteins and under the direction of net absorption [6]. Increased transepithelial Cl^-^ secretion provides a driving force for basolateral-to-apical fluid transport into the intestinal lumen via paracellular pathways leading to net fluid secretion and secretory diarrheas [6]. The cystic fibrosis transmembrane conductance regulator (CFTR), a cAMP-dependent Cl^-^ channel localized to the luminal membrane of enterocytes, is proposed as a promising therapeutic target for secretory diarrheas [7,8]. The CFTR-mediated Cl^-^ secretion is achieved through cooperative functions of the Na^+^-K^+^-2Cl^-^ cotransporters (NKCC1) for uptake of Cl^-^ into enterocytes, and Na^+^/K^+^ ATPase and basolateral cAMP-activated K^+^ channel (KCNQ1/KCNE3) for sustaining its electrochemical driving force [6,9–11]. Apart from CFTR, Ca^2+^-activated Cl^-^ channels (CaCCs) expressed on apical membrane of enterocytes contribute to the pathogenesis of secretory diarrheas especially those involving elevation of intracellular Ca^2+^ such as rotavirus infection [5].

Natural compounds represent a great source of chemicals for identifying potential drug candidates [12]. To date, several CFTR inhibitors have been identified from natural products including plant-derived compounds e.g. xanthones, tannins, flavonoids, chalcones, and piperine, and fungus-derived compounds e.g. zearalenone and arthropsolide A [13–19]. During the last two decades, thousands of microbe-derived bioactive compounds were mostly isolated from fungi [20,21]. Many primary and secondary metabolites produced from soil fungi serve as potential sources of pharmaceutical products, for example, alkaloids, pigments, antibiotics, enzymes, value-added dairy products, and lipid-lowering statins [22]. One of the promising approaches for drug discovery for neglected diseases is drug repurposing. Statins, FDA-approval drugs used for the treatment of dyslipidemia, are originally isolated from fungi. This study was aimed to identify new candidate anti-diarrheal agents from the collection of metabolites from soil fungi and characterize their cellular mechanisms, potential toxicity and anti-diarrheal efficacy using both *in vitro* and *in vivo* models.

## 2. Materials and methods

### 2.1 Ethics statement

All animal experiments were approved by the Institutional Animal Care and Use Committee of the Faculty of Science, Mahidol University (permit number MUSC63-007-515), which have been conducted in accordance with the Guide for the Care and Use of Laboratory Animals of the National Institutes of Health, U.S.A.

### 2.2 Materials

α,β-dehydrolovastatin (DHLV) and its derivatives, lovastatin and α,β-dehydrodihydromonacolin K, were produced by the soil-derived fungus *Aspergillus sclerotiorum* PSU-RSPG178, which was deposited as BCC56851 at BIOTEC Culture collection, National Center for Genetic Engineering and Biotechnology (BIOTEC), Thailand [23]. Mevastatin, pravastatin, and simvastatin (Cat# M2537, P4498, and S6196) were purchased from Sigma-Aldrich (St. Louis, MO, USA). Dulbecco’s Modified Eagle Medium/Nutrient Mixture F-12 (DMEM-F12; Cat#12400–024), fetal bovine serum (FBS; Cat#10270–106), trypsin-EDTA (Cat#25300–062), penicillin/streptomycin (Cat#15140–122) were purchased from Thermo Fisher Scientifc Inc. (Waltham, MA, USA). Cholera toxin (CT; Cat#10654, Lot#10067A1) was obtained from List Biological Laboratories, Inc. (Campbell, CA, USA). Heat-stable toxin (STa; Product no.4044297, Lot#1000007536) was purchased from Bachem (Torrance, CA, USA). Dorsomophin or Compound C (AMPK inhibitor; Cat#P5499), CPT-cAMP (Cat#C3912), genistein (Cat#C6649), ATP (Cat#A5394), forskolin (Cat#F6886), Na_3_VO_4_ (Cat#S6508), CFTR_inh_-172 (Cat#C2992), IBMX (phosphodiesterase (PDE) inhibitor; Cat#I5879), Ouabain (Cat#O3125) were purchased from Sigma-Aldrich (St. Louis, MO, USA). MK571 (Cat# 70720) was purchased from Cayman Chemical (Michigan, USA). Other chemicals were purchased from Merck Millipore (Burlington, MA, USA).

### 2.3 Cell culture

T84 cells were purchased from the American Type Culture Collection (Manassas, VA, USA). The culture medium for this cell line was DMEM-F12 supplemented with 10% FBS and 100 U/mL penicillin, and 100 μg/mL streptomycin. T84 cells were cultured at 37°C in a humidified incubator under an atmosphere of 95% O_2_/5% CO_2_.

### 2.4 Cell viability assays

The cytotoxic effect of DHLV was evaluated by the 3-(4,5-dimethyl-2-thiazolyl)-2,5-diphenyl-2H-tetrazolium bromide (MTT) assays as described previously [24] Briefly, T84 cells were seeded (1 x 10^5^ cells/well) and cultured for 24 h on 96-well plates, followed by treatment for 6 h or 24 h with serum free culture media containing DMSO as a vehicle control or DHLV at various concentrations. MTT reagent (5 mg/mL) was added into 96-well plates containing T84 cells and incubated for 4 h at 37°C. DMSO (100 μL) was added into each well to stop MTT reaction. An absorbance at 540 nm was detected using a spectrophotometer.

### 2.5 Barrier function measurements

Evaluation of the barrier function of intestinal epithelial cell monolayers was performed by measuring the transepithelial electrical resistance (TER) using an epithelial volt-ohm meter (World Precision Instruments, Sarasota, Florida, USA) and permeability assays. In brief, T84 cells were seeded (5 x 10^5^ cells/well) on Transwell permeable support (Cat#3460; Costar, Cambridge, MA, USA) and cultured for 10 days, when TER was more than 1,000 Ω cm^2^. The *in vitro* permeability assay was performed using fluorescein isothiocyanate (FITC)-labeled dextran (molecular weight of ∼4 kDa). T84 cell monolayers were treated for 6 h with vehicle (DMSO), DHLV (0.5 μM—50 μM), or EGTA (positive control; 3 mM). Then, FITC-dextran was added to the apical chamber at the final concentration of 1 mg/mL. An hour later, the basolateral media were collected for measurement of fluorescence intensity (excitation wavelengths of 485 nm and emission wavelengths of 530 nm) using the multi-mode microplate reader (Biotek, Life Science, Inc., USA). FITC-dextran concentrations were calculated using the standard curve of fluorescence intensity at various FITC-dextran concentrations [25].

### 2.6 Electrophysiological analyses

T84 cells were seeded (5 x 10^5^ cells/well) on Snapwell inserts (Cat#3801; Corning, Inc., 0.4 μm pore polyester membrane) and cultured for 10 days (TER >1,000 Ω cm^2^). Snapwell inserts containing T84 cell monolayers were mounted in Ussing chambers filled with Kreb’s solution (pH 7.4) containing 120 mM NaCl, 25 mM NaHCO_3_, 3.3 mM KH_2_PO_4_, 0.8 mM K_2_HPO_4_, 1.2 mM MgCl_2_, 1.2 mM CaCl_2_, and 10 mM glucose. For apical Cl^-^ current analyses, Cl^-^ gradient solutions were used to create the basolateral-to-apical Cl^-^ gradient. The basolateral high Cl^-^ solution contained 130 mM NaCl, 2.7 mM KCl, 1.5 mM KH_2_PO_4_, 1 mM CaCl_2_, 0.5 mM MgCl_2_, 10 mM HEPES (pH 7.4), and 10 mM glucose. The apical low Cl^-^ solution contained 65 mM NaCl were replaced with 65 mM of Na gluconate and the concentration of CaCl_2_ was increased to 2 mM. Basolateral membrane permeabilization was done by pre-incubation with amphotericin B (250 μg/mL) for 30 min. The I_SC_ and apical I_Cl^-^_ were measured using DVC-1000 voltage-clamp (World Precision Instruments, USA and Physiologic Instruments, USA) with Ag/AgCl electrodes and 3 M KCl agar bridges.

### 2.7 Intracellular cAMP measurement

Intracellular cAMP levels were measured using the cAMP Parameter Assay Kit (Cat# KGE002B, R&D Systems, Minneapolis, Minnesota, USA). T84 cells were seeded (1 x 10^6^ cells/well) on 24-well plates (Cat#3524, Corning, Inc.) and cultured for 24 h. Then, cells were washed with PBS for 3 times followed by 1-h treatment with DMSO (vehicle), DHLV (20 μM), forskolin (20 μM), or forskolin (20 μM) plus DHLV (20 μM) before washing with cold phosphate-buffered saline (PBS) and lysis with cell lysis buffers. Intracellular cAMP was competed with horseradish peroxidase (HRP)-cAMP conjugate for binding on anti-cAMP antibodies. The optical density (O.D.) were measured using the multi-mode microplate reader (Biotek, Life Science, Inc., USA). Intracellular cAMP levels were calculated using a standard curve of standard cAMP levels.

### 2.8 Intracellular calcium measurement

Intracellular Ca^2+^ levels were measured using the Fluo-8 Calcium Flux Assay Kit (Cat#ab112129, Abcam plc, CB2, 0AX, UK). T84 cells were seeded (1 x 10^5^ cells/well) and cultured for 24 h on 96-black well plates before incubation with Fluo-8 dye-loading solution at 37°C for 30 min and room temperature for 30 min. Cells were then incubated with DMSO (vehicle) or 20 μM of DHLV, followed by fluorescence intensity measurement (excitation wavelengths of 490 nm and emission wavelengths of 525 nm) using the multi-mode microplate reader (Biotek, Life Science, Inc., USA).

### 2.9 Mouse closed-loop models of cholera toxin (CT)-induced diarrheas

ICR mice (30–35 g) were purchased from Nomura Siam International Co., Ltd., Thailand. Mice were acclimated under controlled conditions for 3 days (temperature 22 ± 1°C; relative humidity 30–70%; 12-h dark/light cycle) with access to food and water ad libitum. To investigate the *in vivo* efficacy of DHLV on CT-induced intestinal fluid secretion, mice were fasted for 24 h before experiments. Mice were anesthetized with an intraperitoneal injection of thiopental (50 mg/kg), abdominal incision was made, and ileal closed-loops (length of 2–3 cm) were created by ligation. For intestinal fluid secretion, ileal closed-loops were instilled with 100 μl of sterile PBS containing CT (1 μg/loop) with or without intraluminal (i.l.) or intraperitoneal (i.p.) administration of DHLV (20 μM and 2 mg/kg, respectively). The abdominal incision was closed by sutures and mice were allowed to recover from anesthesia. Six hours later, mice were euthanized and ileal closed-loops were collected. Weight/length ratios of ileal closed-loops, which indicated intestinal fluid secretion, were measured. For baseline intestinal fluid transport, ileal closed-loops were instilled with 200 μl of PBS with or without DHLV (20 μM). One and thirty min later, mice were euthanized by intraperitoneal injection of overdose of thiopental (150 mg/kg) and ileal closed-loops were collected and measured for weight/length ratios. Mice were euthanized with an injection of thiopental (150 mg/kg).

### 2.10 Statistical analysis

All results are expressed as mean ± S.E.M. Statistical analyses between two groups were performed using Student’s *t*-test. One-way ANOVA was used to compare the difference between three or more groups followed by Bonferroni’s post hoc test. A *p*-value of <0.05 was considered statistically significant.

## 3. Results

### 3.1 Discovery of α,β-dehydrolovastatin (DHLV) as an inhibitor of cAMP-dependent Cl^-^ secretion in T84 cells

**Fig 1:**
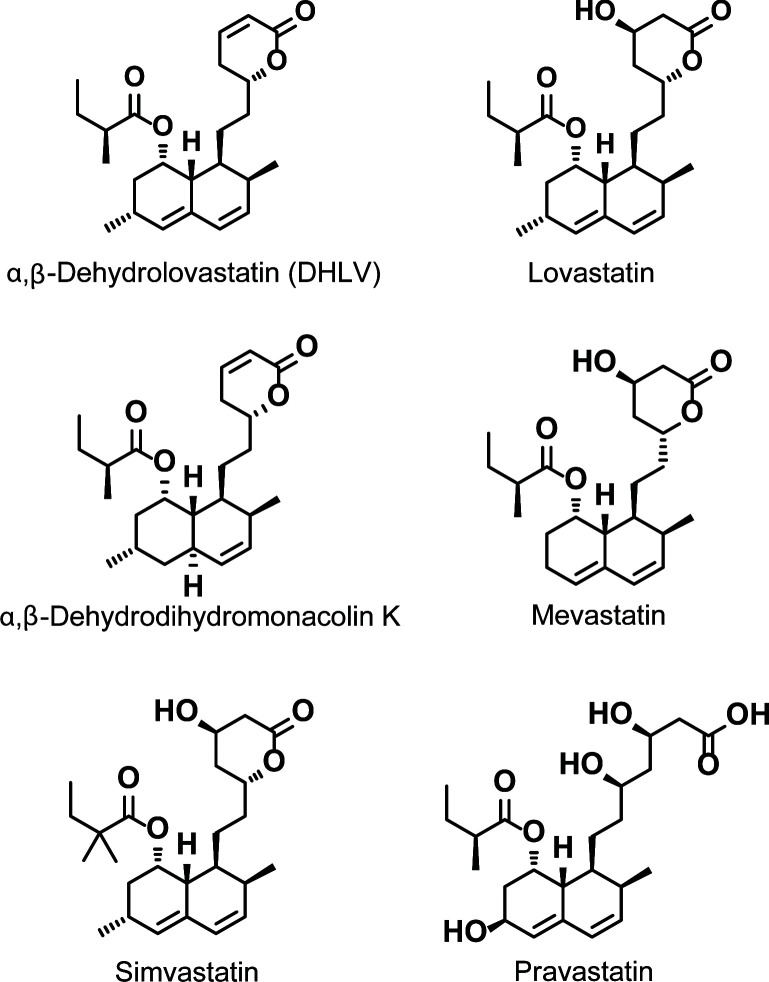
Chemical structures of α,β-dehydrolovastatin (DHLV), lovastatin, α,β-dehydrodihydromonacolin K, mevastatin, simvastatin, and pravastatin.

**Fig 2:**
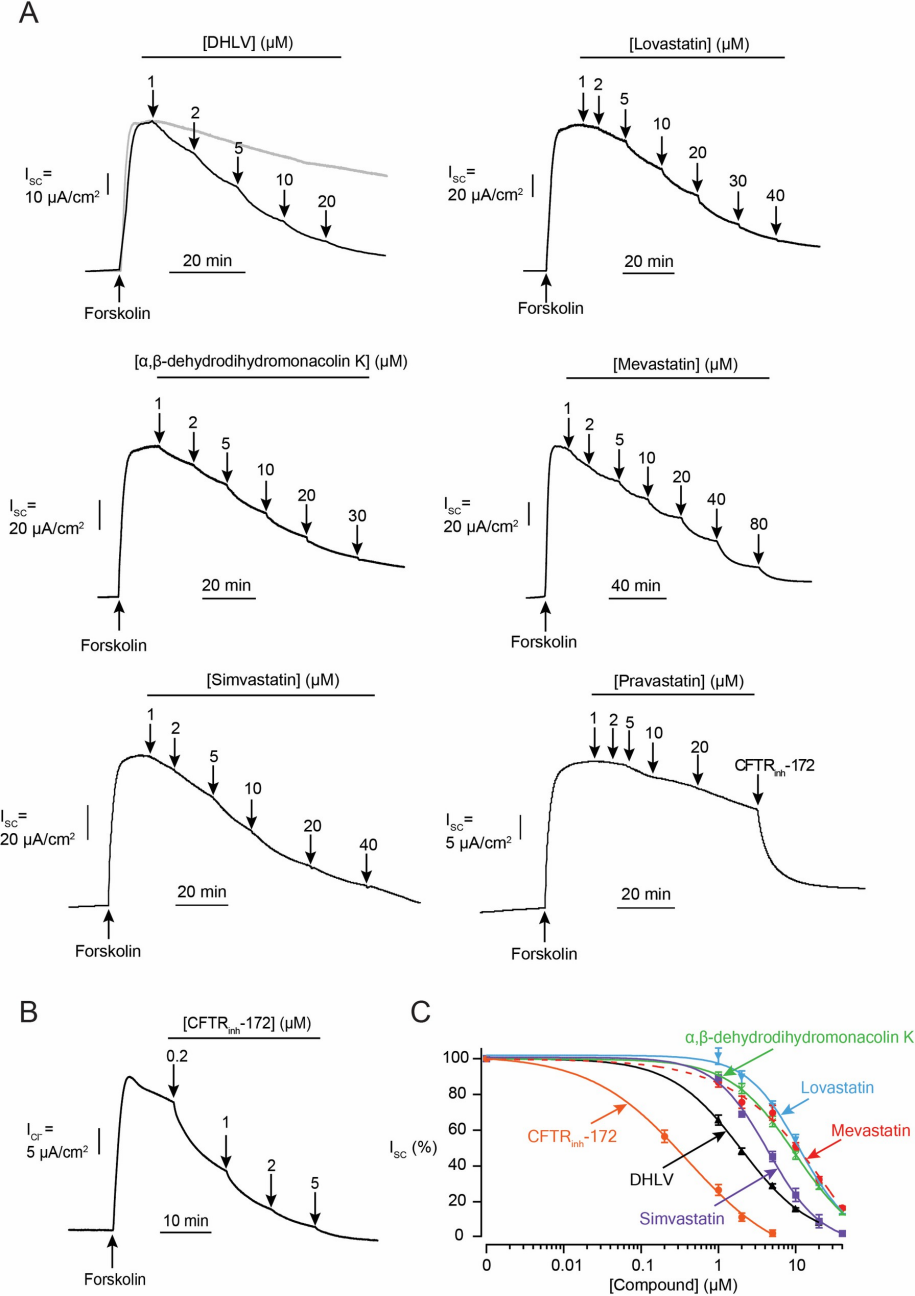
Effect of statin derivatives isolated from soil fungus *Aspergillus sclerotiorum* and statin drugs on cAMP-dependent Cl^-^ secretion in T84 cell monolayers. (A) Effect of α,β-dehydrolovastatin (DHLV), lovastatin, α,β-dehydrodihydromonacolin K, mevastatin, simvastatin, and pravastatin on cAMP-dependent Cl^-^ secretion determined by I_SC_ analysis. (B) Effect of CFTR_inh_-172 (CFTR inhibitor) on cAMP-dependent Cl^-^ secretion determined by I_SC_ analysis. All of compounds were added accumulatively in both apical and basolateral solutions at the indicated concentrations. Representative I_SC_ tracings are shown. (C) Summary of concentration- inhibition studies. Data are fitted to Hill’s equation and expressed as means of % forskolin-stimulated I_SC_ ± S.E.M. (*n* = 3–7).

To identify inhibitors of cAMP-dependent intestinal Cl^-^ secretion, effects of forty metabolites (5 μM) derived from soil fungi on short-circuit current (I_SC_) induced by forskolin (an adenylate cyclase activator) were investigated in T84 cell monolayers [18]. Three statin derivatives isolated from the soil-derived fungus *Aspergillus sclerotiorum* PSU-RSPG178 including α,β-dehydrolovastatin (DHLV), lovastatin, and α,β-dehydrodihydromonacolin K (Fig 1) were active in our assays. Concentration-dependent effects of the three compounds added into both apical and basolateral sides were performed, revealing IC_50_ values of 1.78 ± 0.17 μM, 11.91 ± 1.45 μM and 8.89 ± 1.03 μM, respectively (Fig 2) with maximal inhibitory effects being observed at concentration of 20 μM to 40 μM. Since these compounds shared core chemical structures with the cholesterol-lowering statin drugs, we asked whether other statin drugs (i.e. simvastatin, pravastatin and mevastatin; structures shown in Fig 1) would have inhibitory effects on cAMP- dependent Cl^-^ secretion. We found that simvastatin and mevastatin inhibited cAMP- dependent Cl^-^ secretion with IC_50_ values of 5.03 ± 0.47 μM and 11.39 ± 1.61 μM, respectively (Fig 2A and 2C). In contrast, pravastatin at concentrations between 1 μM to 20 μM did not affect cAMP- dependent Cl¯ secretion. These data indicate that DHLV is more potent than the tested statin drugs and others and less potent than a reference CFTR inhibitor CFTR_inh_-172 (IC_50_ = ∼0.2 μM, Fig 2B) in inhibiting cAMP-dependent Cl¯ secretion in T84 cells. Therefore, subsequent investigations were performed to evaluate pharmacological properties of DHLV including polarity of effects, potential cytotoxicity, mechanism of actions and anti-diarrheal efficacy.

### 3.2 Polarity of inhibition and cytotoxic effects of DHLV

**Fig 3:**
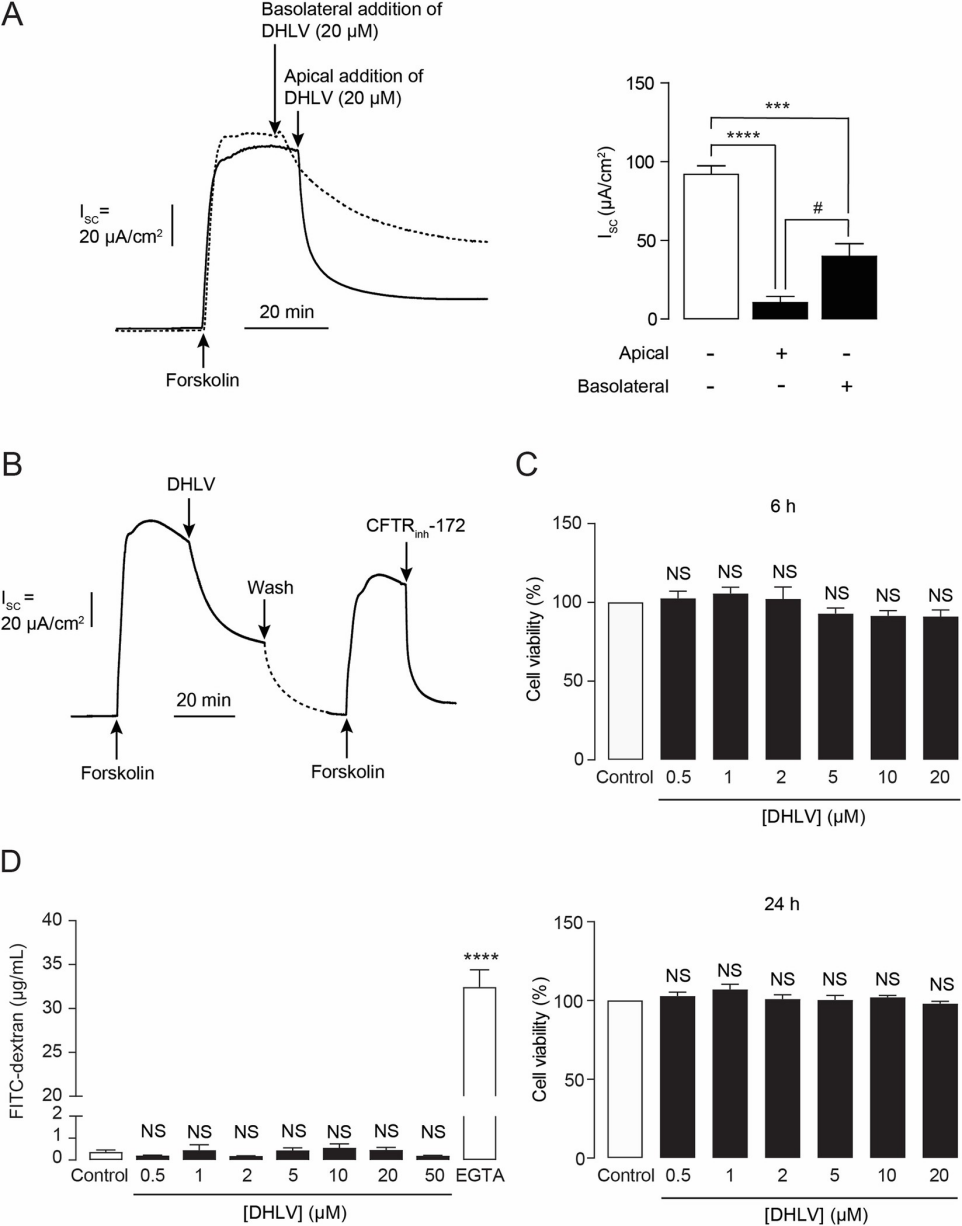
Effect of α,β-dehydrolovastatin (DHLV) on cAMP-dependent Cl^-^ secretion, cell viability and barrier function in T84 cell monolayers. (A, left) Polarity of inhibition by α,β-dehydrolovastatin (DHLV) on cAMP-dependent Cl^-^ secretion determined by I_SC_ analysis. DHLV (20 μM) was added into apical and basolateral solutions in a separate experiment. Representative I_SC_ tracings are shown. (A, right) Summary of the data expressed as mean of I_SC_ ± S.E.M. (*n* = 5). (B) Reversibility of inhibition of CFTR-mediated Cl^-^ secretion by DHLV (2 μM) in T84 cell monolayers. After the inhibition of CFTR-mediated Cl^-^ secretion was stabilized, the bathing solution containing forskolin and DHLV was removed. Both chambers were gently washed 5 times and bathing solution containing forskolin were re-filled into the chamber. At the end of experiment, CFTR_inh_-172 (20 μM) was added into the apical chamber. A representative tracing of 3 independent experiments is shown (*n = 3*) (C) Effect of DHLV on cell viability evaluated by MTT assays. T84 cells were treated with DHLV at the indicated concentrations for 6 or 24 h (upper, lower). Data are expressed as % of cell viability compared to control ± S.E.M. (*n* = 4–6). (D) Effect of DHLV on intestinal barrier function. FITC-dextran flux assays were performed after 6 h of incubation with DHLV (20 μM). EGTA (3 mM) was used as a positive control. Data are expressed as concentration of FITC-dextran ± S.E.M. (*n* = 5–6). NS, non-significant; **** *p* < 0.0001 compared with control.

To investigate the polarity of DHLV inhibition of cAMP-dependent Cl^-^ secretion, effects of DHLV added into apical vs basolateral solutions were compared. DHLV was tested at a concentration of 20 μM, which was found to inhibit cAMP-dependent Cl^-^ secretion by ~95% in the concentration-response studies. As depicted in Fig 3A, basolateral addition of DHLV (20 μM) diminished cAMP-dependent Cl^-^ secretion by ~57.32% in intact T84 cells. On the other hand, apical addition of DHLV produced ~89.1% inhibition of cAMP-dependent Cl^-^ secretion, which was significantly higher than that produced by basolateral addition of DHLV. These results indicate that DHLV preferentially acts on apical membrane.

Next, the reversibility of the inhibitory effect of DHLV was determined. As shown in Fig 3B, removal of DHLV (2 μM) from the bathing solutions resulted in ~60% reversal of the cAMP-dependent Cl^-^ secretion. Of note, the recovered cAMP-dependent Cl^-^ current was inhibited by CFTR_inh_-172, confirming that the recovered current was mainly mediated by CFTR. This result indicates that the inhibitory effect of DHLV on cAMP-dependent Cl^-^ secretion is reversible.

To examine the potential cytotoxic effects of DHLV in T84 cells, MTT cell viability assays and fluorescein isothiocyanate (FITC)-dextran (molecular weight of 4 kDa) permeability assays were performed. Exposure of T84 cells to DHLV (up to 20 μM) for 6 h (exposure time of *in vivo* studies) or 24 h did not affect cell viability of T84 cells as measured by MTT assays (Fig 3C). Similarly, DHLV at concentrations up to 50 μM (6 h of incubation) had no effect on FITC-dextran flux (Fig 3D), indicating that DHLV had no effect on barrier integrity in T84 cell monolayers. These results indicate that DHLV has no potential cytotoxic effect in T84 cells.

### 3.3 Mechanism of DHLV on the inhibition of cAMP-dependent Cl^-^ secretion

**Fig 4:**
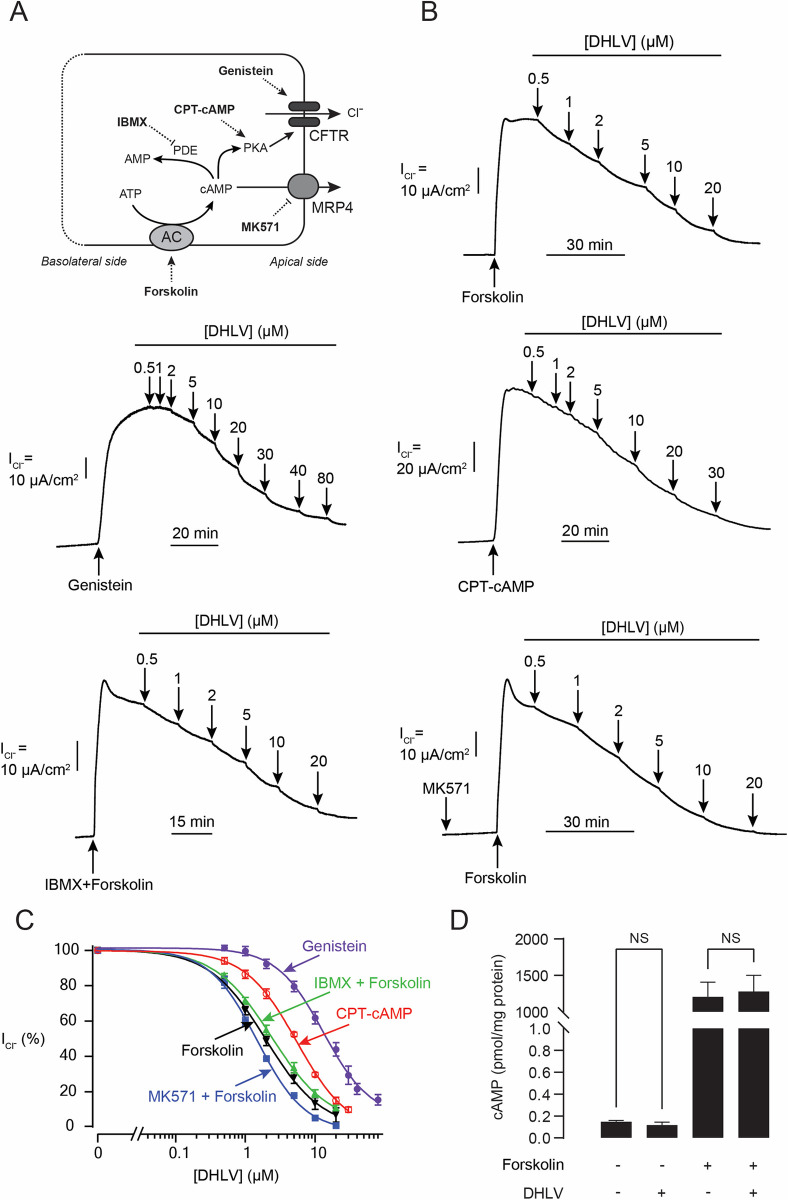
Mechanism of α,β-dehydrolovastatin (DHLV) actions on cAMP-dependent CFTR activity in intestinal epithelial T84 cell monolayers. (A) Schematic diagrams showing the regulatory mechanism of CFTR Cl^-^ channel activity. (B) Effect of DHLV on cAMP-dependent Cl^-^ secretion determined by apical I_Cl^-^_ analysis induced by forskolin (20 μM), genistein (20 μM), or CPT-cAMP (100 μM). The involvement of phosphodiesterase (PDE) and multidrug-resistance 4 (MRP4) was determined by 15 min pre-treatment with IBMX (1 mM) or MK571 (20 μM), respectively, prior to forskolin treatment. Representative I_SC_ tracings are shown. (C) Summary of concentration-inhibition studies. Data are fitted to Hill’s equation and expressed as means of % agonist-stimulated I_Cl^-^_ ± S.E.M. (*n* = 3–7). (D) Effect of DHLV on intracellular cAMP levels. T84 cells were pre-treated with vehicle (control) or DHLV (20 μM) for an hour. Intracellular cAMP levels were measured by cAMP parameter assay kits. Data are expressed as concentrations of cAMP ± S.E.M. (*n* = 3). NS, non-significant compared with indicated group.

To scrutinize whether DHLV inhibited the CFTR Cl^-^ transport activity, apical Cl^-^ current (I_Cl¯_) analysis was performed. In this experiment, basolateral membrane of T84 cell monolayers was permeabilized by amphotericin B (250 μg/mL) and buffers with asymmetrical Cl^-^ concentrations were used to establish a basolateral-to-apical Cl^-^ gradient (Fig 4A). Using forskolin as a CFTR stimulator, DHLV concentration-dependently inhibited the CFTR-mediated apical I_Cl^-^_ with an IC_50_ value of 2.10 ± 0.25 μM and >95% inhibition being observed at a concentration of 20 μM (Fig 4B and 4C). Interestingly, we found that DHLV inhibited apical I_Cl^-^_ induced by genistein (a direct activator of CFTR) with an IC_50_ value of 9.92 ± 1.86 μM, which was significantly higher than that obtained from experiments using forskolin (an indirect activator of CFTR) as a stimulator of CFTR-mediated apical I_Cl¯_ (Fig 4B and 4C).

Next, we determined if DHLV inhibited the CFTR-mediated apical I_Cl^-^_ by modulating intracellular cAMP metabolism. We found that DHLV suppressed the CFTR-mediated apical I_Cl^-^_ induced by CPT-cAMP (a non-hydrolysable, cell-permeable cAMP) and a cocktail of forskolin and IBMX with IC_50_ values of 5.50 ± 0.21 μM and 2.59 ± 0.36 μM, respectively (Fig 4B and 4C). Apart from global cAMP regulation by PDE, multidrug-resistant protein 4 (MRP4)-mediated cAMP efflux controls CFTR activity by modulating local cAMP levels in a compartmentalized manner [26]. To evaluate the role of MRP4 in mediating the inhibitory effect of DHLV on CFTR, T84 cells were pretreated with MK571, an inhibitor of MRP4, before conducting concentration-dependent CFTR inhibition studies of DHLV. In the presence of MK571, we found that DHLV inhibited the forskolin-induced CFTR-mediated apical I_Cl^-^_ with an IC_50_ value of 1.66 ± 0.32 μM (Fig 4B and 4C). Furthermore, effects of DHLV on intracellular cAMP levels were investigated. DHLV at a concentration of 20 μM did not affect intracellular cAMP levels under both basal and forskolin-stimulated conditions (Fig 4D). All together, these results indicate that the mechanism of CFTR inhibition by DHLV does not involves alteration in intracellular cAMP levels or stimulation of either PDE or MRP4.

**Fig 5:**
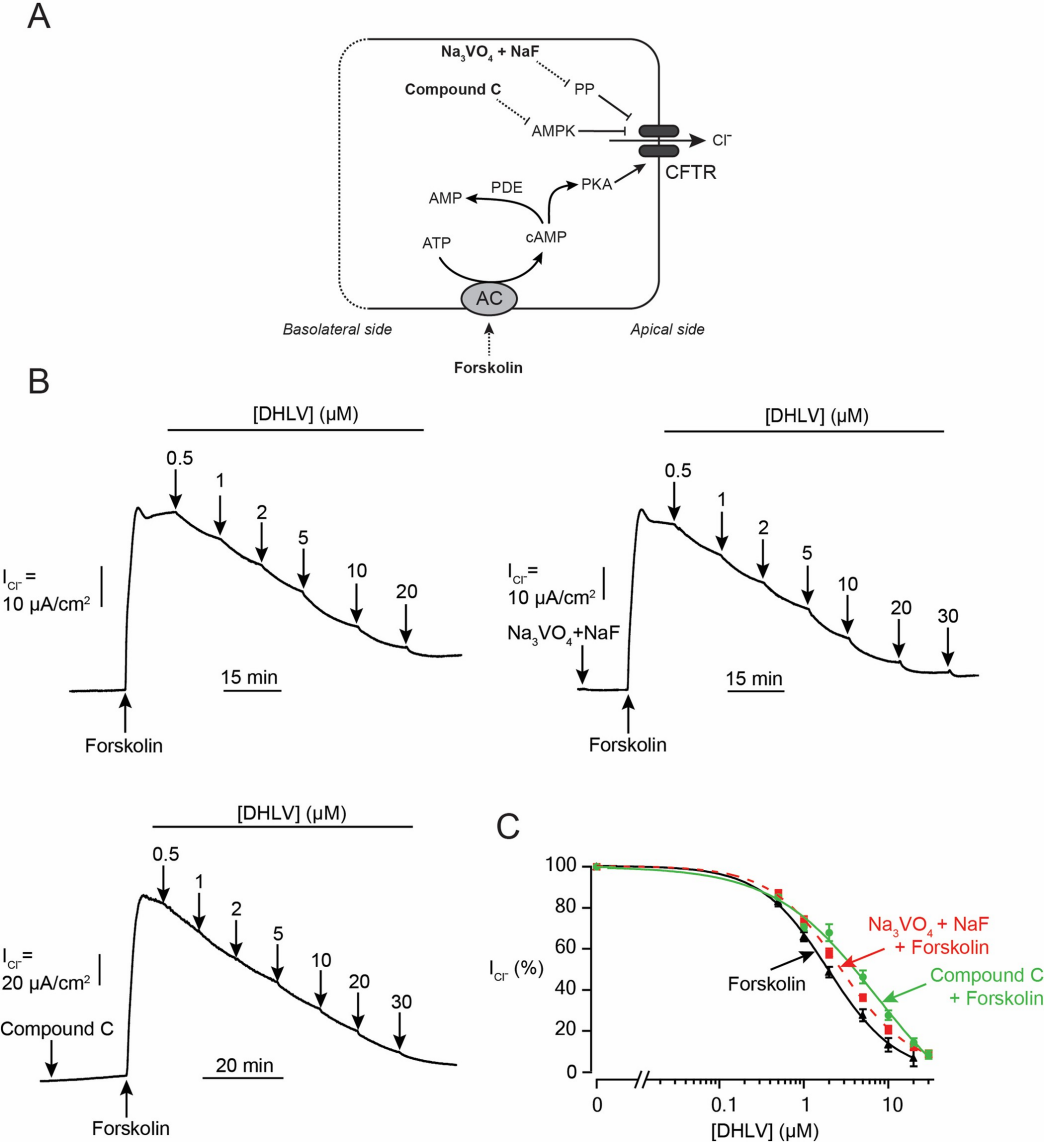
No involvement of CFTR negative regulators in CFTR inhibition by DHLV. (A) Schematic diagrams showing the regulatory mechanism of CFTR Cl^-^ channel activity. (B) apical Cl^-^ current (I_Cl^-^_) tracing showing the effect of α,β-dehydrolovastatin (DHLV) on forskolin-induced CFTR Cl^-^ secretion in T84 cells pre-treatment with compound C (50 μM) or and NaF plus Na_3_VO_4_ (1 mM) for 15 min. (C) Summary of concentration-inhibition studies. Data are fitted to Hill’s equation and expressed as means of % agonist-stimulated I_Cl^-^_ ± S.E.M. (*n* = 5–9).

Protein phosphatases (PP) and AMP-activated protein kinases (AMPK) are negative regulators of CFTR activity [27,28]. We next asked whether DHLV inhibited CFTR via these two proteins (Fig 5A). We found that pretreatment of T84 cells with Na_3_VO_4_ and NaF (a cocktail of PP inhibitors) or compound C (AMPK inhibitor) had no effect on IC_50_ values of DHLV obtained from the dose-response studies using forskolin as a CFTR activator (IC_50_ values of 2.71 ± 0.26 μM and 4.45 ± 0.44 μM vs 2.10 ± 0.25 μM, respectively) (Fig 5B and 5C). These results indicate that CFTR inhibition by DHLV is independent of AMPK and PP activities.

### 3.4 Effects of DHLV on Ca^2+^-activated Cl^-^ channels (CaCC) and basolateral Na^+^/K^+^ ATPases

**Fig 6:**
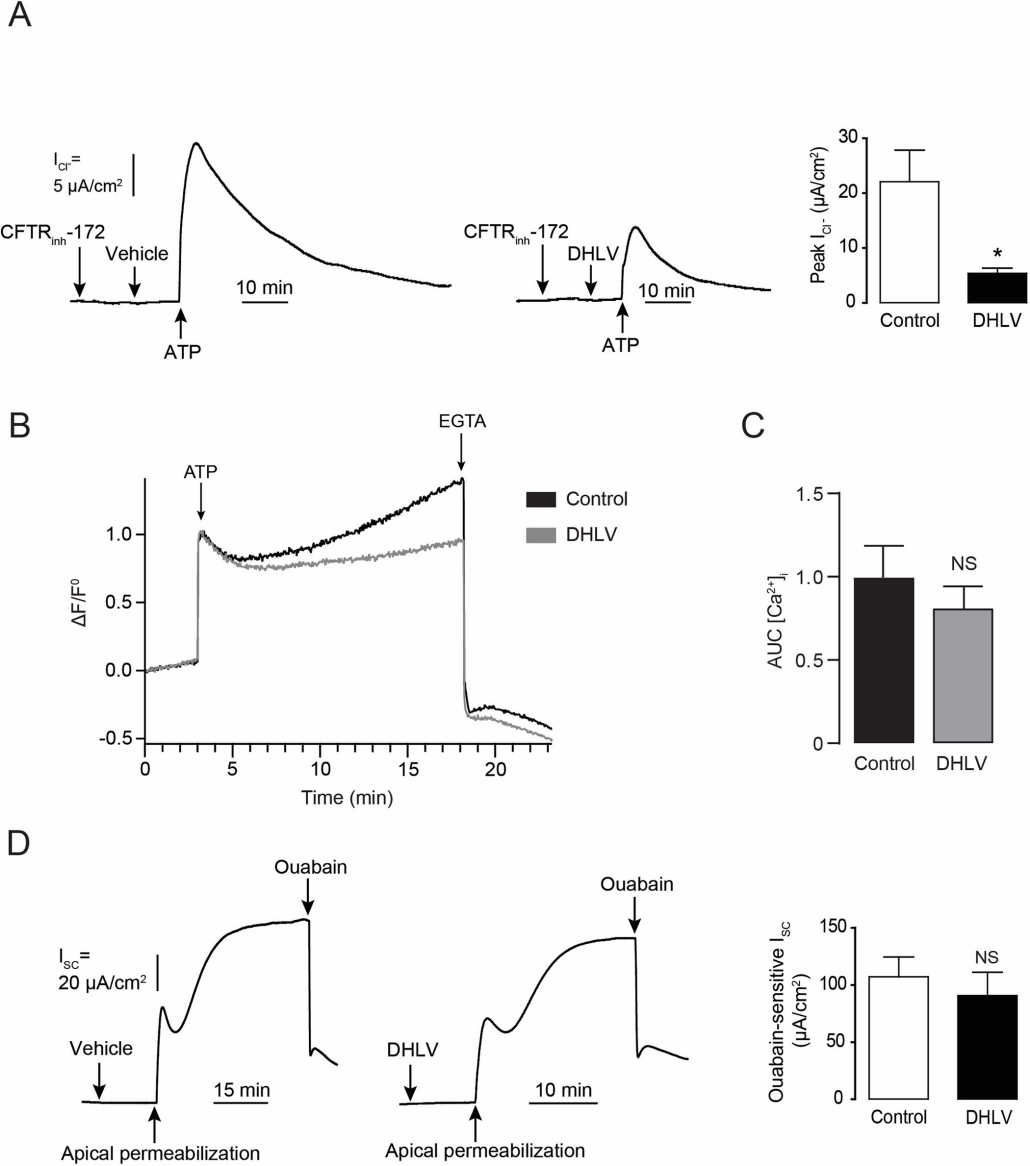
Effects of α,β-dehydrolovastatin (DHLV) on Ca^2+^-activated Cl^-^ channels (CaCC), the intracellular Ca^2+^ levels induced by ATP and Na^+^/K^+^ ATPases activity. (A, left) Representative apical I_Cl^-^_ tracing showing the effect of ATP (100 μM) activation after 15 min pre-treatment with CFTR_inh_-172 (5 μM) with vehicle (control) or DHLV (20 μM). (A, right) Summary of the data of peak I_Cl^-^_ expressed as mean of peak I_Cl^-^_ ± S.E.M. (*n* = 5). * *p* < 0.05 compared with control. Intracellular Ca^2+^ levels were analyzed from the fluo-8-based fluorescence assays. Fluo-8 fluorescence intensity were measured following vehicle (control) or DHLV (20 μM) together with the addition of ATP (100 μM) and end up with EGTA (3 mM). (B) Representative values of the fractional change in fluorescence intensity relative to baseline (ΔF/F^0^) are shown. (C) Summary of total AUC values after baseline correction presented as the area under the curve (AUC) ± S.E.M (*n* = 4). NS, non-significant (Student’s t test). (D, left) Ouabain-sensitive I_SC_ tracing showing the effect of DHLV on Na^+^-K^+^ ATPase activity. After T84 cells were permeabilized at apical membrane, ouabain (1 mM) was added. (D, right) Summary of the data are expressed as mean of ouabain-sensitive I_SC_ ± S.E.M. (*n* = 4). NS, non-significant (Student’s t test).

In addition to CFTR, CaCC provides a principal route for chloride secretion especially in response to elevation of intracellular Ca^2+^ levels. We next determined effects of DHLV on CaCC in T84 cells using apical I_Cl^-^_ analyses. In this experiment, CaCC-mediated apical I_Cl^-^_ was induced by application of ATP following a pretreatment with CFTR_inh_-172, a CFTR inhibitor, to prevent contribution of CFTR [17]. Peak of ATP-induced CaCC-mediated apical I_Cl^-^_ was significantly reduced by ~75% with the DHLV (20 μM) pretreatment (Fig 6A). We also determined the immediate effect of DHLV on intracellular Ca^2+^ levels using Fluo-8 assay kits. DHLV had no significant effects on both ATP-induced intracellular Ca^2+^ elevation as analyzed by area under the curve (AUC) (Fig 6B and 6C). These results indicate that mechanisms of CaCC inhibition by DHLV do not involve alternation of intracellular Ca^2+^ levels. Since basolateral Na^+^/K^+^ ATPases are important for maintaining driving force of intestinal Cl^-^ secretion, we tested whether DHLV affected Na^+^/K^+^ ATPase activity. In this experiment, Na^+^ loading following apical membrane permeabilization stimulated Na^+^/K^+^ ATPase activity reflected by an increase in I_SC_. Na^+^/K^+^ ATPase activity was quantified from the values of I_SC_ inhibited by ouabain, an inhibitor of Na^+^/K^+^ ATPase. As shown in Fig 6D, DHLV (20 μM) did not affect ouabain-sensitive I_SC_, suggesting that DHLV had on effect on Na^+^/K^+^ ATPase activity.

### 3.5 Antidiarrheal effect of DHLV in a mouse model of enterotoxin-induced intestinal fluid secretion

**Fig 7:**
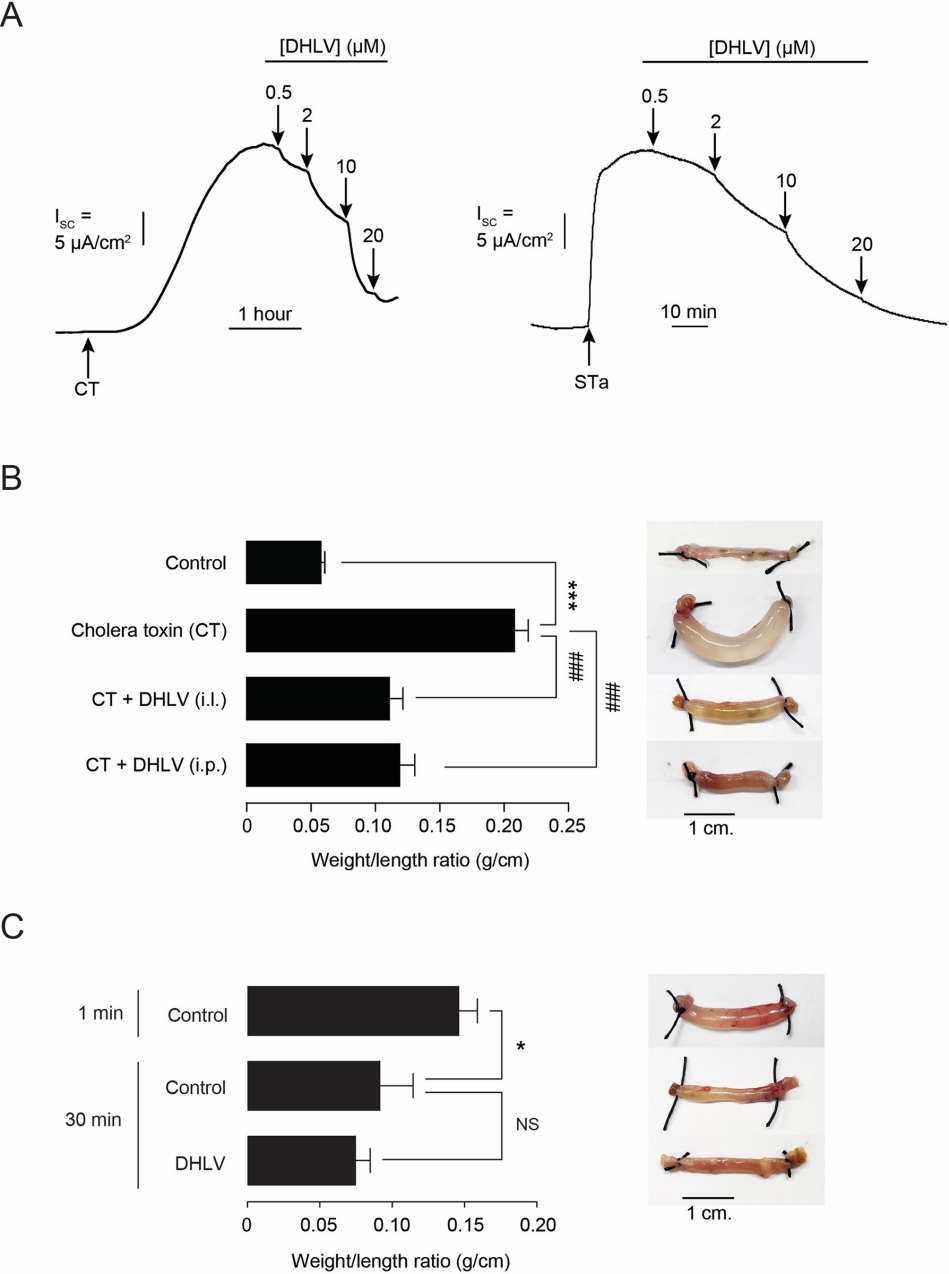
Potential utility of α,β-dehydrolovastatin (DHLV) as an anti-diarrheal agent for secretory diarrheas. (A) Effect of DHLV on cholera toxin (CT)- and heat-stable toxin (STa)-stimulated Cl^-^ secretion in T84 cell monolayers determined by I_SC_ analysis. After apical addition of CT (1 μg/ml) or STa (100 μM) to stimulate the increasing of I_SC_, DHLV was added in both apical and basolateral solution. Representative I_SC_ tracings are shown. (B) Effect of DHLV on CT-induced intestinal fluid secretion determined by ileal closed-loop weight/length ratios. Ileal closed-loops were injected with PBS (control) or PBS containing CT (μg/loop) with or without intraluminal (i.l.) and intraperitoneal (i.p.) administrations of DHLV (20 μM and 2 mg/kg, respectively). Representative photographs of ileal loops are shown. Summary of data are expressed as means of ileal closed-loop weight/length ratio ± S.E.M. (*n* = 5–6). *** *p* < 0.001 compared with control; ### *p* < 0.001 compared with CT-treated group (one-way ANOVA). (C) Effect of DHLV on the net intestinal fluid transport determined by ileal closed- loop weight/length ratios. Ileal closed-loop were injected with PBS (200 μl) with or without intraluminal administrations of DHLV (20 μM). After 30 min, ileal closed-loop weight/length ratio were measured. Representative photographs of ileal closed-loops are shown. Summary of data are expressed as means of ileal closed-loop weight/length ratio ± S.E.M. (*n* = 6–7). * *p* < 0.05 compared with control at 1 min; NS, non-significant compared with control at the same time point (one-way ANOVA).

Bacterial enterotoxins including cholera toxin (CT) and heat-stable toxin (STa) are major virulence factors responsible for intestinal fluid secretion and fluid loss in secretory diarrheas [5]. To determine potential utility of DHLV in the treatment of secretory diarrheas, effects of DHLV on enterotoxin-induced transepithelial Cl^-^ secretion and fluid secretion were determined in T84 cell monolayers and mice, respectively. As depicted in Fig 7A, DHLV inhibited both CT- and STa-induced I_SC_ in a concentration-dependent manner with >90% inhibition was observed at a concentration of 20 μM. To evaluate *in vivo* efficacy of DHLV, ileal closed-loop model of CT-induced fluid secretion was performed in mice. We found that intraluminal (20 μM) and intraperitoneal (2 mg/kg) administrations of DHLV significantly reduced CT-induced fluid secretion analyzed from loop weight/length ratios by ∼65% and ∼59%, respectively (Fig 7B). Furthermore, effects of DHLV on net baseline fluid transport were assessed using ileal closed-loop models in mice. As shown in Fig 7C, at 30 min after instilling PBS into ileal loops, fluid was absorbed by ~40% as analyzed from the weight/length ratios, which was unaffected by intraluminal administration of DHLV (20 μM). These results suggest that DHLV inhibits CT-induced intestinal fluid secretion without affecting net baseline intestinal fluid transport.

## 4. Discussion

In the present study, we revealed a novel effect of statin derivatives obtained from fungi and in clinical use on inhibiting cAMP-dependent Cl^-^ secretion across human intestinal epithelial (T84) cells. Electrophysiological analyses indicate that α,β-dehydrolovastatin (DHLV), a statin derivative from the soil-derived fungus *Aspergillus sclerotiorum* PSU-RSPG178, is the most potent derivative inhibiting CFTR-mediated Cl^-^ secretion via mechanisms not involving negative regulators of CFTR functions including AMPK and protein phosphatases, or alteration of intracellular cAMP levels. In addition, CaCC-mediated Cl^-^ secretion was inhibited by DHLV. Importantly, DHLV attenuated diarrheal severity by suppressing intestinal fluid secretion in the mouse model of CT-induced diarrhea without affecting basal intestinal fluid transport.

Based on the observation that the addition of DHLV into apical solutions produced the maximal inhibition of cAMP-dependent Cl^-^ secretion, whereas addition of DHLV into basolateral solutions partially reduced cAMP-dependent Cl^-^ secretion, indicating that DHLV targeted CFTR at the apical membrane of T84 cells. Of note, pharmacokinetic profile of DHLV is not currently available. It is possible that DHLV may be transported across the cellular membrane via active transport. Indeed, absorption of some statins (i.e., pravastatin) was reported to be transported into intracellular sites of small intestine via the organic anion transporting polypeptide (OATP-B), which is located on apical membrane [29]. Therefore, it is possible that DHLV might also be transported via OATP-B into intestinal epithelial cells, which accounts for apical polarity of DHLV’s effect.

MTT assays indicate that DHLV has no cytotoxic effect on T84 cells. In addition to serving as a physical barrier, tight junction-dependent intestinal barrier function establishes cell polarity, which is known to support vectoral transport across intestinal epithelial cells [30–32]. FITC-dextran (4 kDa) permeability assays indicate that intestinal barrier function is not affected by DHLV treatment. Moreover, DHLV did not inhibit Na^+^-K^+^ ATPase activity, which is required for the regulation of intracellular Na^+^ and cell volume in intestinal epithelial cells [33,34]. Based on our findings, CFTR-inhibiting effect of DHLV is not associated with overt toxicity to intestinal epithelial cells, which is one of prerequisite properties of an antidiarrheal therapy [35].

The inhibitory effect of DHLV on CFTR-mediated Cl^-^ secretion induced by forskolin was more potent than that induced by genistein. Tyrosine kinases and protein phosphatases are known to regulate the activity of CFTR chloride channel by phosphorylation and dephosphorylation at nucleotide-binding domains 1 and 2 (NBD1 and NBD2), respectively [36,37]. Genistein stimulates CFTR directly by interacting with CFTR and indirectly by inhibiting tyrosine kinases and protein phosphatases [38]. Therefore, we speculate that the reduction of DHLV potency in inhibiting genistein-induced CFTR-mediated Cl^-^ secretion may be due to the reduced potency of DHLV to inhibit CFTR activity induced by tyrosine kinase and/or protein phosphatase inhibition.

Overproduction of cAMP is stimulated by CT through stimulation of a signaling cascade involving adenylyl cyclase and protein kinase A (PKA), which stimulates intestinal fluid secretion and accounts for pathogenesis of secretory diarrheas [39]. In addition, inhibition of PDE or MRP4, the regulators of intracellular cAMP levels, leads to stimulation of transepithelial Cl^-^ secretion and causes secretory diarrheas [26,40]. It is possible that DHLV decreases the levels of cAMP, which results in inhibition of CFTR-mediated Cl^-^ secretion [41]. However, our results showed that DHLV inhibited CFTR activity without the contribution of PDE and MRP4. In addition, DHLV had no effect on the intracellular cAMP levels. Therefore, we speculate that mechanisms of CFTR inhibition by DHLV may result from its direct actions on CFTR channel activity or its indirect actions on other proteins regulating CFTR function.

AMPK has been reported to inhibit CFTR activity and limit cAMP-dependent Cl^-^ secretion, which effectively decreases intestinal fluid secretion in acute diarrheal illness [42,43]. This regulatory role of AMPK is supported by the evidence showing that AMPK activation is involved in the anti-secretory mechanism of some natural compounds [16]. However, we found that pretreatment with AMPK inhibitor did not alter the inhibitory effect of DHLV on CFTR activity. In fact, statins were reported to rapidly activate AMPK via increased Thr-172 phosphorylation in vascular endothelial cells [44]. Since other mechanisms may contribute to the action of DHLV, we further investigated whether protein phosphatases, another negative regulator of CFTR activity, are a target of DHLV action. Protein phosphatases are able to dephosphorylates R domain of CFTR and cause deactivation of CFTR [27]. Indeed, our findings showed that pretreatment with protein phosphatase inhibitors did not affect the ability of DHLV to inhibit CFTR activity, suggesting that protein phosphatases are not involved in the inhibitory effect of DHLV on CFTR function.

It is accepted that important properties of potential anti-diarrheal therapy include *in vivo* anti-diarrheal efficacy and no interference on intestinal fluid absorption [45]. Indeed, DHLV inhibits both CFTR and CaCC-mediated Cl^-^ secretion, implying that DHLV may be beneficial in the treatment of secretory diarrheas caused by stimulation of either cAMP or Ca^2+^. *In vitro* studies using CT and STa toxins as activators of Cl¯ secretion in T84 cells showed that DHLV effectively inhibited prosecretory effects of both toxins. Since STa induces CFTR-mediated Cl^-^ secretion via a cGMP-dependent mechanism [46], DHLV appears to inhibit intestinal Cl^-^ secretion elicited by three main second messengers. It is noteworthy that anti-secretory efficacy of DHLV given via intraperitoneal (2 mg/kg) or intraluminal (20 μM) route in mice is ∼59.30% and ∼64.64%, respectively. At the baseline condition, measurement of fluid transport showed that DHLV had no effect on net intestinal fluid transport in mice. This dose (2 mg/kg) of DHLV is equivalent to 0.16 mg/kg in humans, which is in the dose range of statin drugs in the treatment of dyslipidemia [47]. The route of medication depends on the drug’s properties [48]. Based on our findings in mice in this study, it is possible that DHLV exerts their anti-secretory action in diarrheal patients via intraluminal administration route, which is easier to apply and associated with potentially less systemic side effects or toxicities compared to intravenous administration [48]. Our results indicate that DHLV inhibits intestinal fluid secretion by inhibiting CFTR and CaCC channels. Limitation of this study include the lacking of investigations on effects of DHLV on other transporters involved in intestinal fluid transport and pharmacokinetics and safety profiles of DHLV, which warrants further studies.

## 5. Conclusion

In summary, α,β-dehydrolovastatin (DHLV) and other statins represent a novel class of inhibitors of intestinal apical Cl¯ channels including CFTR and CaCC, which are involved in the pathogenesis of severe diarrheas including cholera. Although oral rehydration therapy is highly effective, there is no drug therapy approved for cholera aside from broad spectrum antibiotics. Further research and development on DHLV may lead to the successful development of inexpensive and effective treatment of cholera.
